# Cathelicidin- derived PR39 protects enterohemorrhagic *Escherichia coli* O157:H7 challenged mice by improving epithelial function and balancing the microbiota in the intestine

**DOI:** 10.1038/s41598-019-45913-6

**Published:** 2019-07-01

**Authors:** Zhang Haiwen, Hua Rui, Zhang Bingxi, Guan Qingfeng, Wang Beibei, Zeng Jifeng, Wang Xuemei, Wu Kebang

**Affiliations:** 1 Tropical animal breeding and nutrition laboratory, Hainan University, Haikou, Hainan, 570228 People’s Republic of China; 2 Key Laboratory of Tropical Biological Resources of Ministry of Education, Haikou, Hainan, 570228 People’s Republic of China

**Keywords:** Acute inflammation, Bacterial infection

## Abstract

The zoonotic enterohaemorrhagic *Escherichia coli* (EHEC) O157:H7 can disrupt intestinal epithelial barrier function and in turn leading to serious intestinal and systemic disease. PR39 could effectively inhibit the growth of Gram-negative bacteria, but there is little knowledge of its effects on intestinal barrier function and the microbiota in *E*. *coli*-challenged mice. In this study, an intestinal disease caused by EHEC O157:H7 was established, to analyze the effect of PR39 on EHEC O157:H7 induced intestinal epithelial barrier injury and disorder. Interestingly, PR39 attenuated EHEC O157:H7-induced systemic symptoms and significantly decreased mortality and the degree of *E*. *coli* shedding in faeces. Furthermore, the infiltration index of macrophages and neutrophils in intestine of the PR39 treatment group were obviously attenuated, along with the level of apoptosis. PR39 treatment group had distinctly improved tight junction associated proteins’ expression after EHEC O157:H7 caused injury. Additionally, the sequencing analysis of cecum microbiota showed that PR39 altered the abnormal increase in *Bacteroides* caused by EHEC O157:H7 and promoted the growth of probiotics such as *Lactobacillus*. In conclusion, cathelicidin-derived PR39 could effectively improve EHEC O157:H7-induced epithelial barrier injury, and dysfunction of immune and microbiota homeostasis in the intestinal tract, indicating that PR39 could be an excellent potential drug for zoonotic EHEC O157:H7-related intestinal disease.

## Introduction

Enterohaemorrhagic *Escherichia coli* (EHEC), as pathogenic subgroup of Shiga toxin-producing *E*. *coli* (STEC)^1^, can produce Shiga toxins 1 and/ or 2 (Stx1 and Stx2) and usually have an adhesion gene (eae). *E*. *coli* O157:H7 is the most common serotype of EHEC^2^, it is also one of the most prevalent serotypes causing diarrhoea in humans, adhering tightly to the intestinal mucosa to form attaching and effacing lesions. Such lesion causes destruction of intestinal microvilli structure and leads to bloody diarrhoea, especially during the infant period^2^. Notably, the Shiga toxin (Stx) secreted by *E*. *coli* O157:H7 can pass through the intestinal epithelium and induce systemic damage^3^, generating inflammatory responses and causing apoptosis of epithelial cells. The Stx will further result in haemolytic uremic syndrome (HUS), which is a major cause of acute kidney injury in children^4^.

It is widely believed that the treatment of EHEC infected individuals with antibiotics would increase the risk of developing HUS^5^. Once EHEC infection is identified, there is no effective drug to reduce the risk of developing HUS disease^6^, so the search for effective and safe therapeutic appears to be urgent^5^. Antimicrobial peptides are short cationic peptides with amphipathic structures, widely distributed among animals and plants, which serve a fundamental role in host defence against pathogens^7^. There are two main families of mammalian antimicrobial peptides, the defensins and the cathelicidins, which are secreted by immune cells and certain epithelial cells^8^. The importance of cathelicidin-derived peptides has been demonstrated on protecting the skin^9^, and urinary^10^ and gastrointestinal tracts^11^ against bacterial infections.

PR39 was initially identified in the homogenate of the small intestine of a pig, and it was the first porcine cathelicidin-derived peptide identified^12^. PR39 has specific antibacterial activity against multiple gram-negative bacteria by inhibiting protein synthesis and unique membrane-disruptive effects^13^. It was also reported that PR39 improved the surface expression of syndecan-1 and syndecan-4 on the mesenchymal cells, which accelerated the healing of wounds^14^. In the mouse model of lipopolysaccharide (LPS)-induced sepsis, PR39 could protect the liver through increasing the production of nitric oxide (NO) in the liver and limiting the generation of reactive oxygen species (ROS)^15^. PR39 also influences polarisation of porcine macrophages, polarising them from a M2 to a M1 phenotype, and promotes the phagocytic function of macrophages^16^.

To date, the effects of the antimicrobial peptide PR39 on EHEC O157:H7-caused systemic disorder and symptoms remain unreported, as does the function of PR39 in recovery from dysbiosis. Therefore, this study explored the effects of PR39 on the inflammatory level, gastrointestinal epithelial function, and intestinal microbiota, using a mouse model of EHEC O157:H7 infection.

## Results

### PR39 attenuated the clinical symptoms of EHEC O157:H7 induced infection

Clinical features caused by EHEC O157:H7 were macroscopic soft shit, listless, no appetite and no desire to to move were observed in EHEC O157:H7 induced group (named group O157 below) compared with other three groups. As shown in Supplemental Fig. S1A, group O157 showed sustained body weight loss in comparison with other three groups, while group PR39 + O157 showed weight lose trend during the period of first inoculation, but since the second inoculation at day 5, the weight gain was improved and showed no significance with group Control check (CK). However, at day 6, compared with group CK, the weight loss of group O157 was significant (p < 0.05), and the weight loss status became even worse (p < 0.01) at day 8 and day 10. During the experimental period, The survival rate of each group were analyzed. Due to severe illness resulted in death of certain mice, the survival rate of group O157 was only 60%, while the group PR39 + O157 reached up to 90% (Supplemental Fig. S1B). For the low survival rate of the group O157, to keep the consistency of each group, the sampling amounts of other groups were only six animals per group. The full spleen tissues and one pair of thymus were collected, to calculate the corresponding organ index, and to evaluate the immune performance of each group indirectly. The spleen index of group CK and PR39 + O157 were significantly higher than group O157 (p < 0.05), while group CK and PR39 + O157 showed no significant difference (Supplemental Fig. S1C). The thymus index showed the same tendency (Supplemental Fig. S1D).

### PR39 improved the intestinal permeability disrupted by EHEC O157:H7

As shown in Supplemental Fig. S2A, since the starting time (0 min) to the end time (60 min), the TEER value of other three groups were significantly higher than group O157 (p < 0.05). The concentration of fluorescein isothiocyanate-dextran (FITC-dextran) in serum was also determined. The serum concentration of FITC-dextran in CK and PR39 + O157 were significantly lower than group O157 (p < 0.05), the concentration in group PR39 + O157 reduced close to group CK (Supplemental Fig. S2B). The concentration of Diamine oxidase and D-lactic acid in serum were also tested. The group O157 showed significant higher concentration than group CK and PR39 + O157 (p < 0.05), consistently in both two indexes (Supplemental Fig. S2C,D), and PR39 administration decreased this two indexes to be closed to the group CK level.

### PR39 reverted the level of serum cytokines of EHEC O157:H7 infected mice

As shown in Supplemental Fig. S3, the concentration of IL-1β and IL-6 in group O157 were significantly higher than group CK and PR39 + O157 (Supplemental Fig. S3A,B) (p < 0.05). In group O157, the level of TNF-α showed even highly significant level than group CK (p < 0.01), while showing significantly higher level than group PR39 + O157 (p < 0.05), though there still existed significance between group CK and PR39 + O157 (Supplemental Fig. S3C) (p < 0.05). The level of IL-10 in CK and PR39 + O157 were significantly higher than group O157 (Supplemental Fig. S3D) (p < 0.05). In contrast, the treatment of PR39 had reversed the abnormal expression level of cytokine in serum induced by EHEC O157:H7.

### PR39 decreased EHEC O157:H7 caused damage to the intestinal morphology

**Figure 1:**
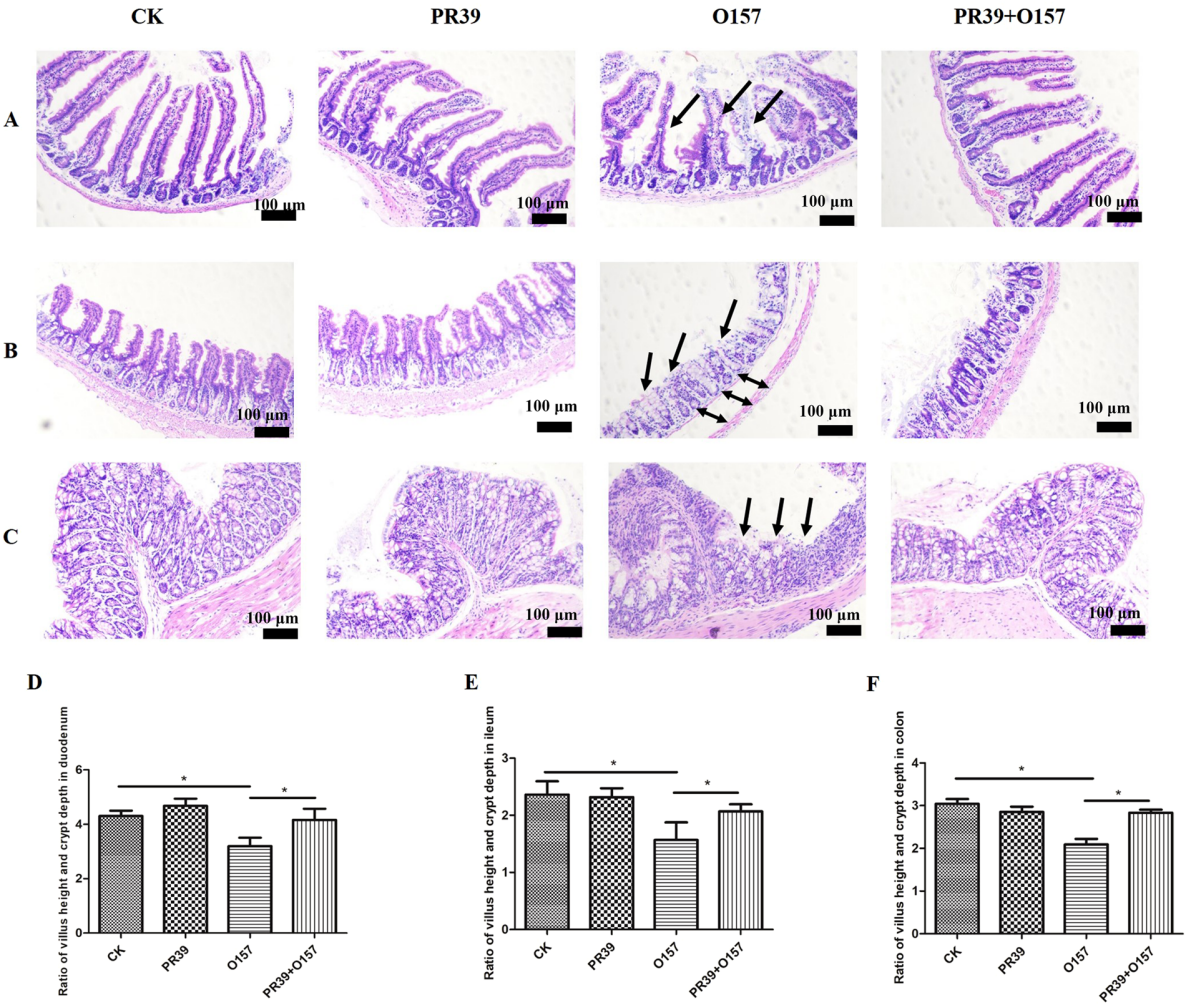
Epithelial morphological observation of duodenum (**A**), ileum (**B**) and colon (**C**) tissues (100×). The value of villous height: crypt depth of duodenum (**D**), ileum (**E**) and colon (**F**). All of the data are expressed as the mean ± SD. *Means p < 0.05, **means p < 0.01.

**Figure 2:**
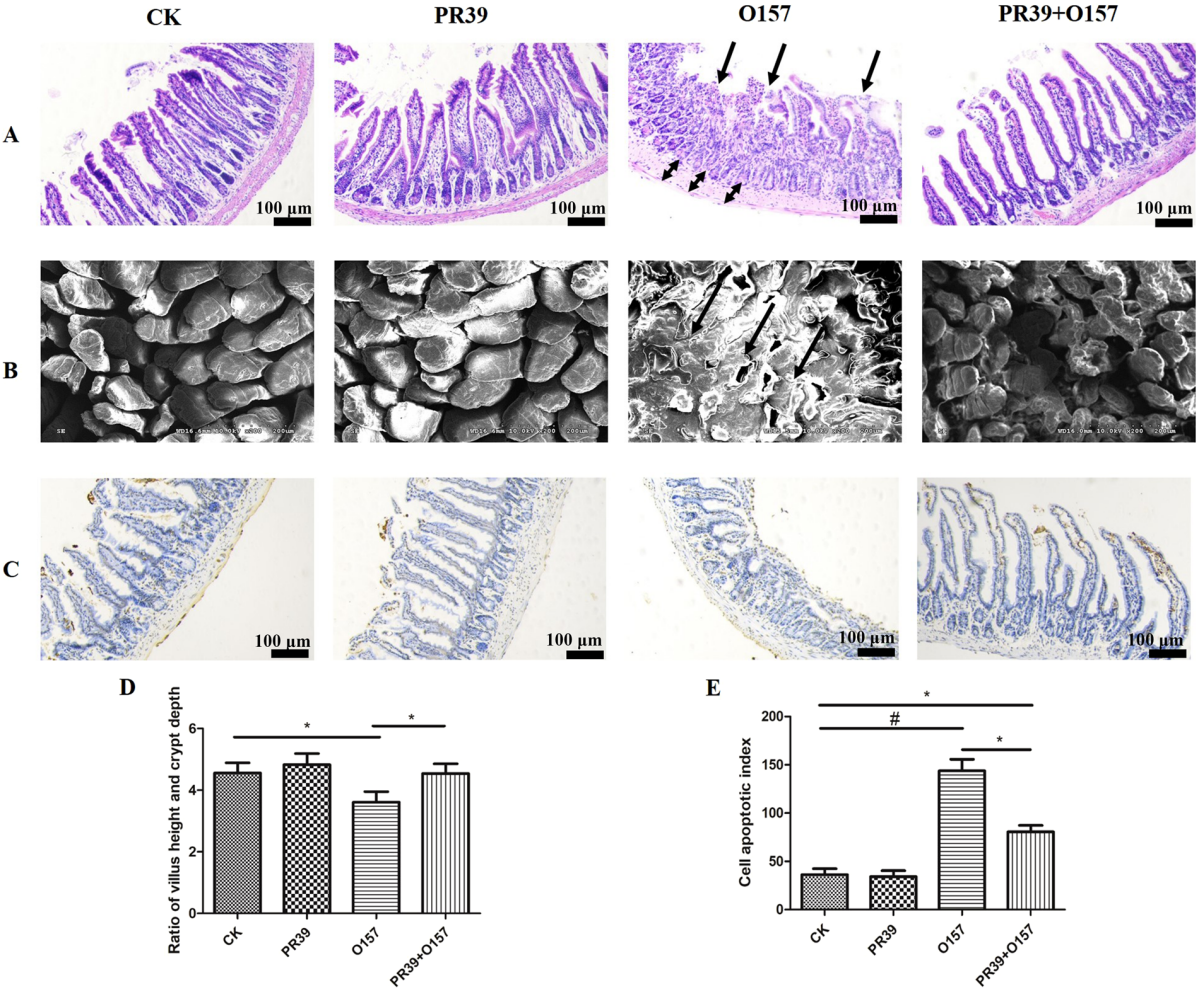
Epithelial morphological observation of jejunum (**A**) tissues (100×). Scanning electron microscope observation of jejunum (200×) (**B**). Apoptosis analysis of jejunum (100×) (**C**). The value of villous height: crypt depth of jejunum (**D**). The cell apoptotic index (**E**). All of the data are expressed as the mean ± SD. *Means p < 0.05, ^#^means p < 0.01 compared with group CK.

As shown in Figs 1A–C and 2A, it could be directly observed that *E*. *coli* O157 caused serious damage to the intestinal segments, with sparse and shortened intestinal villi, damaged structure (indicated as black arrows). Significant edema were observed in the submucosa of the ileum and jejunum (Figs 1B and 2A, indicated as black double arrows). The statistic analysis of the ratio of villus height and crypt depth in each intestinal segments showed consistently that group O157 had significantly lower value of villus height: crypt depth than group CK and PR39 + O157 (Figs 1D,E and 2D) (p < 0.05), while PR39 treatment prominently reversed the trend. Further SEM scanning (Fig. 2B) and TUNEL analysis (Fig. 2C) were applied to analyze the exact microvillus architecture and epithelium apoptosis level in jejunum. Group O157 showed uneven and fuzzy characteristics of microvillus (indicated as black arrows), while PR39 treatment attenuated the symptoms (Fig. 2B). Cell apoptotic index in jejunum epithelium of group O157 showed highly significance level than group CK (p < 0.01), and significantly higher than group PR39 + O157 (p < 0.05), although the apoptotic index in group CK was still significantly lower than group PR39 + O157 (p < 0.05).

### PR39 increased barrier function related proteins’ expression in the jejunum of EHEC O157:H7 infected mice

**Figure 3:**
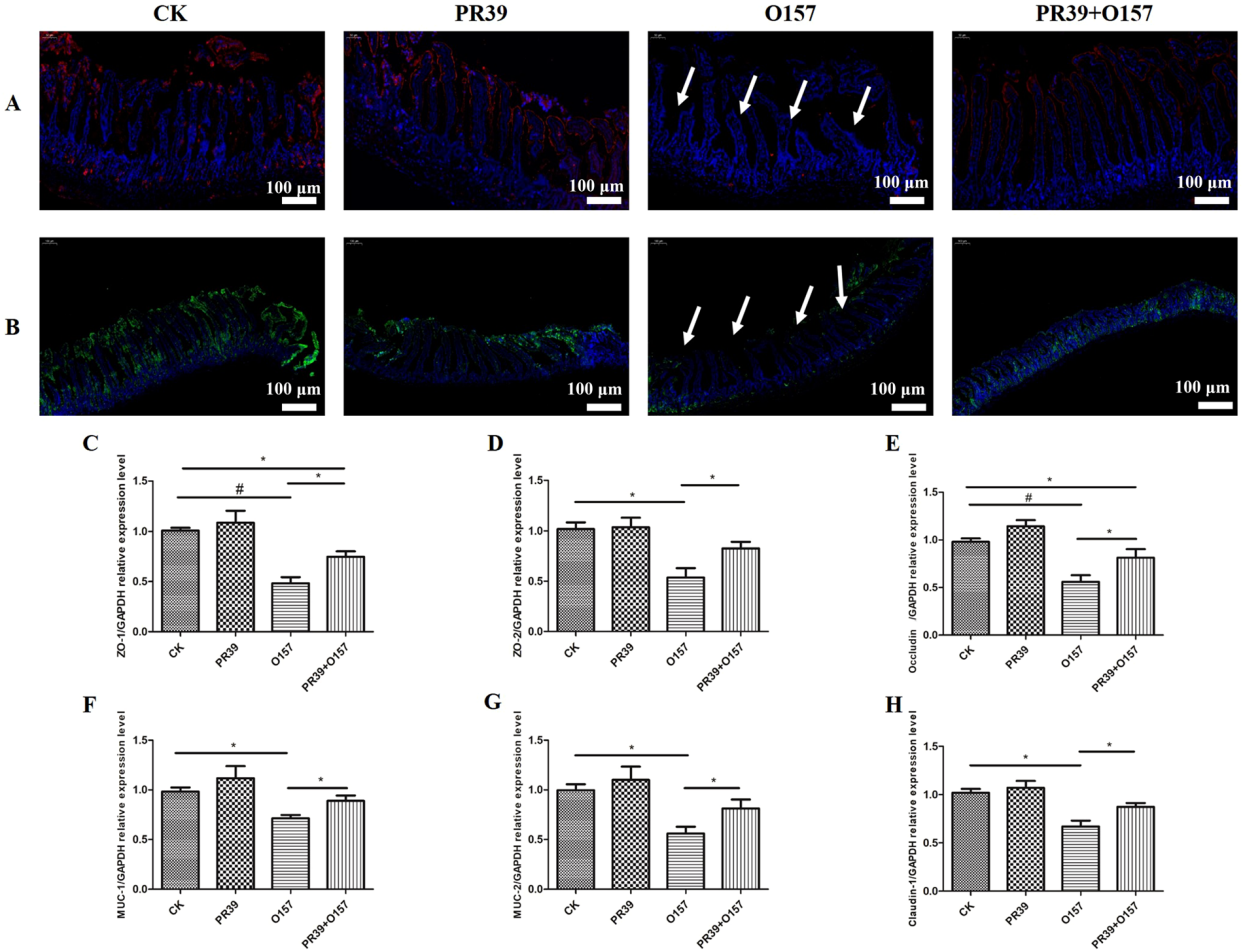
Fluorescence staining analysis of ZO-1(**A**, red color) and occluding (**B**, green color) in jejunum tissues (100×). Relative gene expression of ZO-1 (**C**), ZO-2 (**D**), Occludin (**E**), MUC-1 (**F**), MUC-2 (**G**) and Claudin-1 (**H**) in jejunum. All of the data are expressed as the mean ± SD. *Means p < 0.05, ^#^means p < 0.01 compared with group CK.

**N**The expression level of ZO-1 and Occludin (Fig. 3A,B) in jejunum tissue of group O157 were significantly lower than other three groups (indicated as white arrows). Consistently, the relative gene expression level of tight junction (TJ) proteins of ZO-1 (Fig. 3C) and Occludin (Fig. 3E) showed highly significant difference between group O157 and CK (p < 0.01). The expression level of this two proteins showed significant difference between group O157 and PR39 + O157 (p < 0.05), while there still existed significant difference between group CK and PR39 + O157 (p < 0.05).

### PR39 attenuated the infiltration index of macrophage and neutrophil in intestinal mucosa of jejunum and colon tissues

**Figure 4:**
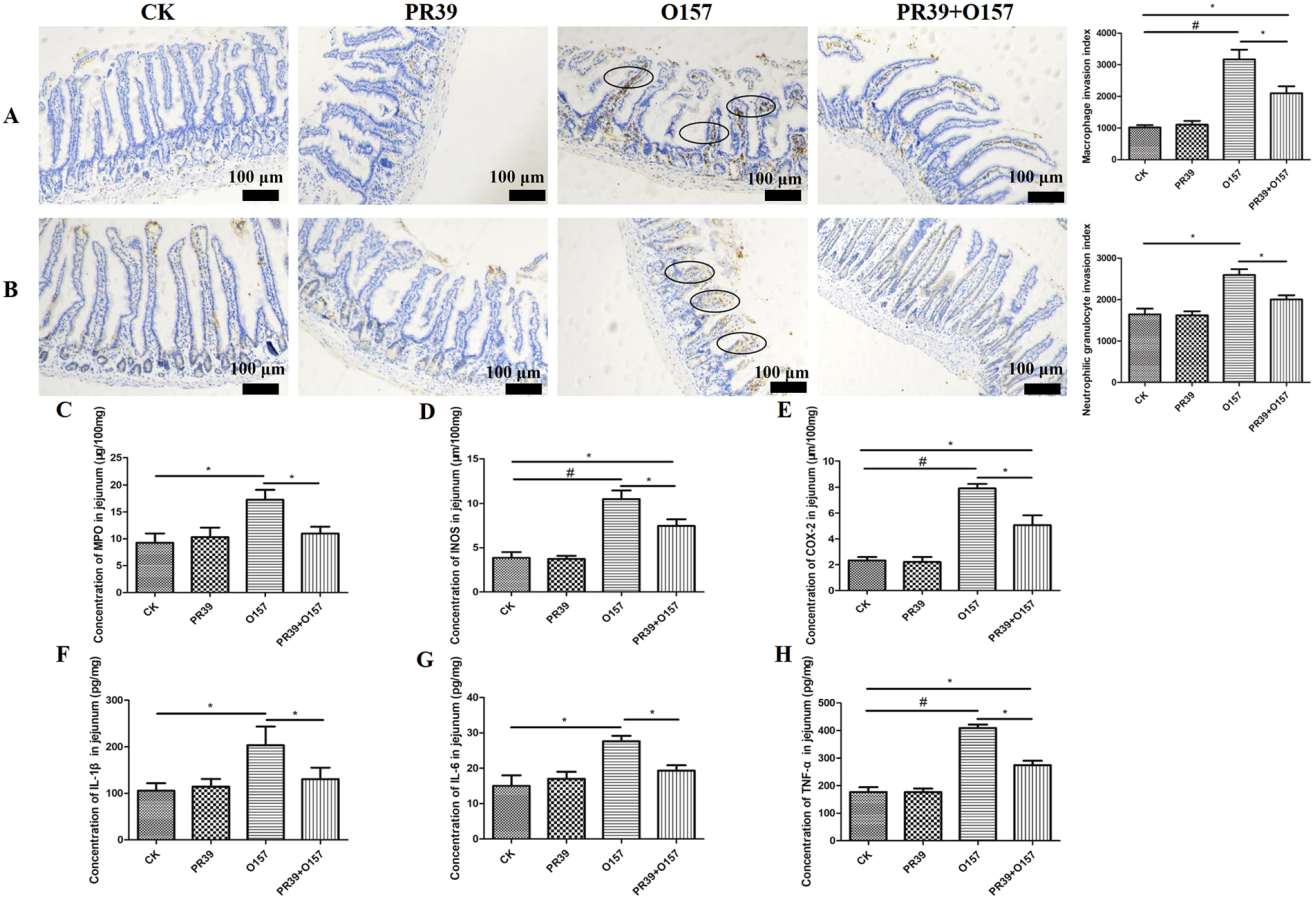
Histochemical staining analysis of macrophages (F4/80) (**A**) and neutrophils (CD177) (**B**) in jejunum tissues and the corresponding invasion index (n = 6 per group). The concentration of inflammation related enzyme MPO (**C**), INOS (**D**), COX-2 (**E**) and pro-inflammatory cytokines IL-1β (**F**), IL-6 (**G**), TNF-α (**H**) in jejunum. All of the data are expressed as the mean ± SD. *Means p < 0.05, ^#^means p < 0.01 compared with group CK.

**Figure 5:**
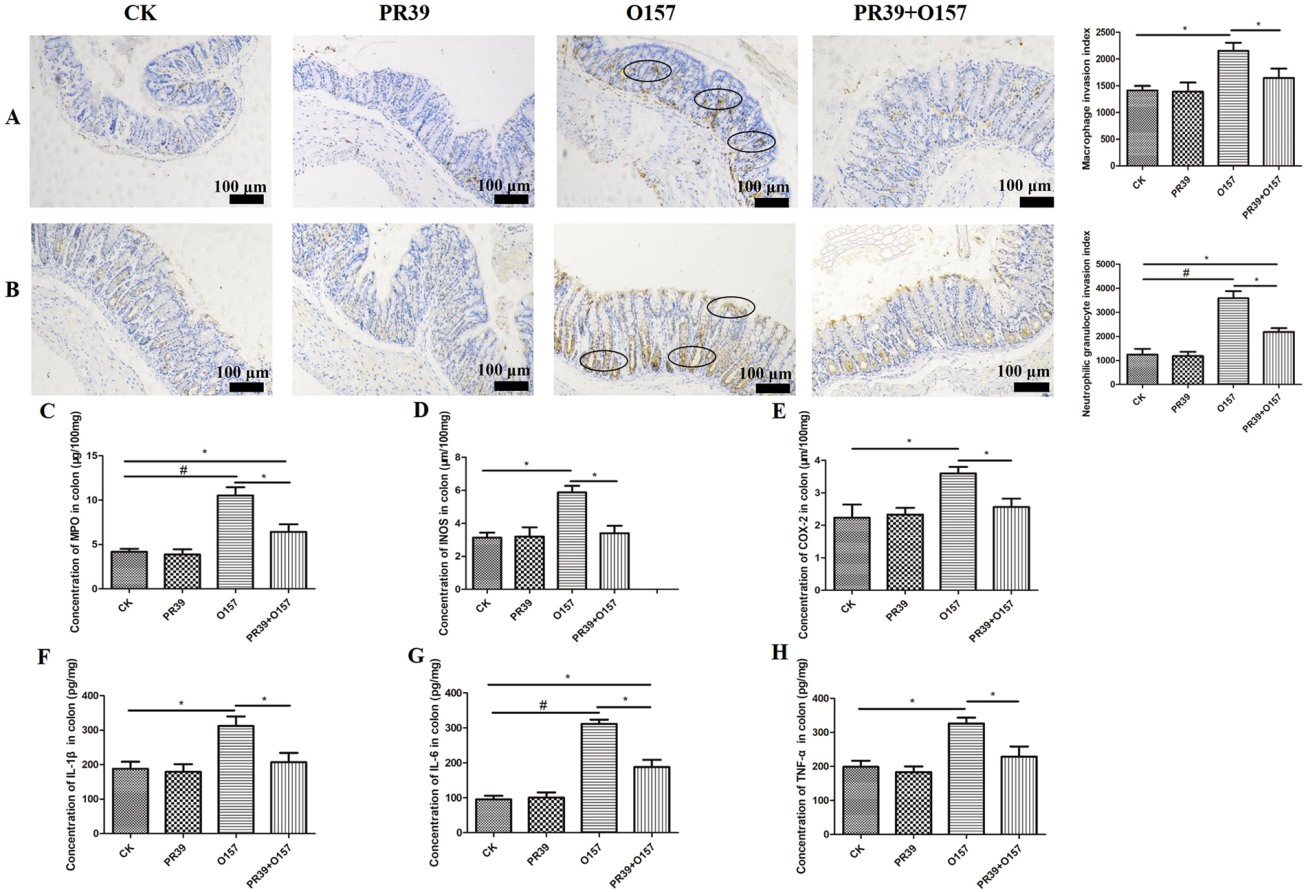
Immunohistochemistry staining of macrophages (F4/80) (**A**) and neutrophils (CD177) (**B**) in colon tissues and the corresponding invasion index (n = 6 per group). The concentration of inflammation related enzyme MPO (**C**), INOS (**D**), COX-2 (**E**) and pro-inflammatory cytokines IL-1β (**F**), IL-6 (**G**), TNF-α (**H**) in colon. All of the data are expressed as the mean ± SD. *Means p < 0.05, ^#^means p < 0.01 compared with group CK.

The specific antibodies against surface marker of macrophages and neutrophils, F4/80 and CD177, were utilized to evaluate the inflammatory infiltration status of jejunum (Fig. 4) and colon (Fig. 5) intestinal mucosa by inflammatory cells. The macrophage invasion index in jejunum and neutrophils invasion index in colon of group O157 (indicated as black oval) showed highly significant difference compared with group CK (p < 0.01). The macrophage and neutrophils invasion index in jejunum and colon of group O157 were significantly higher than group PR39 + O157 (p < 0.05), and the macrophage invasion index in jejunum and neutrophils invasion index in colon of group PR39 + O157 were still significantly higher than group CK (p < 0.05) (Figs 4A and 5B). The neutrophils invasion index in jejunum and macrophage invasion index in colon of group O157 showed significant higher level than both group CK and PR39 + O157 (p < 0.05) (Figs 4B and 5A). The concentration of enzyme myeloperoxidase (MPO), which was specifically secreted by neutrophils, showed the same rule with the corresponding invasion index of neutrophils in jejunum and colon (Figs 4C and 5C). The secretion level of INOS, COX-2, TNF-α in jejunum and IL-6 in colon of group O157 showed highly significant difference compared with group CK (p < 0.01), the comparison between PR39 + O157 and CK, PR39 + O157 and O157 showed significant difference (p < 0.05). Additionally, the secretion level of IL-1β and IL-6 in jejunum and INOS, COX-2, IL-1β, TNF-α in colon of group O157 showed significantly higher level than group CK and PR39 + O157 (p < 0.05).

### PR39 improved the architecture of liver and kidney

**Figure 6:**
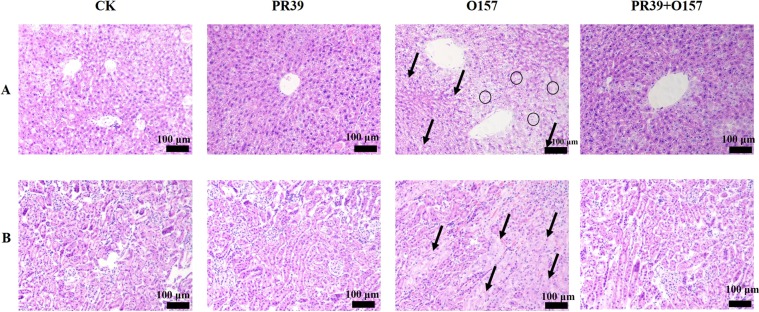
Morphological observation of Liver (**A**) and kindey (**B**) (n = 6 per group) (100×).

The boundary of hepatic lobule in group O157 was not clear, and the hepatic plate arrangement was irregular (indicated as black arrows), many hepatocytes showed vacuolation phase, and the cell nucleus showed atrophic form (indicated as black circles), and the PR39 treatment group obviously improved pathological morphology described above (Fig. 6A). Typical pathological features in kidney were observed such as the blurred boundaries of renal tubules and the obvious apoptosis symptoms (indicated as black arrows), while PR39 treatment reduced kidney damage (Fig. 6B).

### PR39 balanced the bacterial community and SCFAs production in cecum

**Figure 7:**
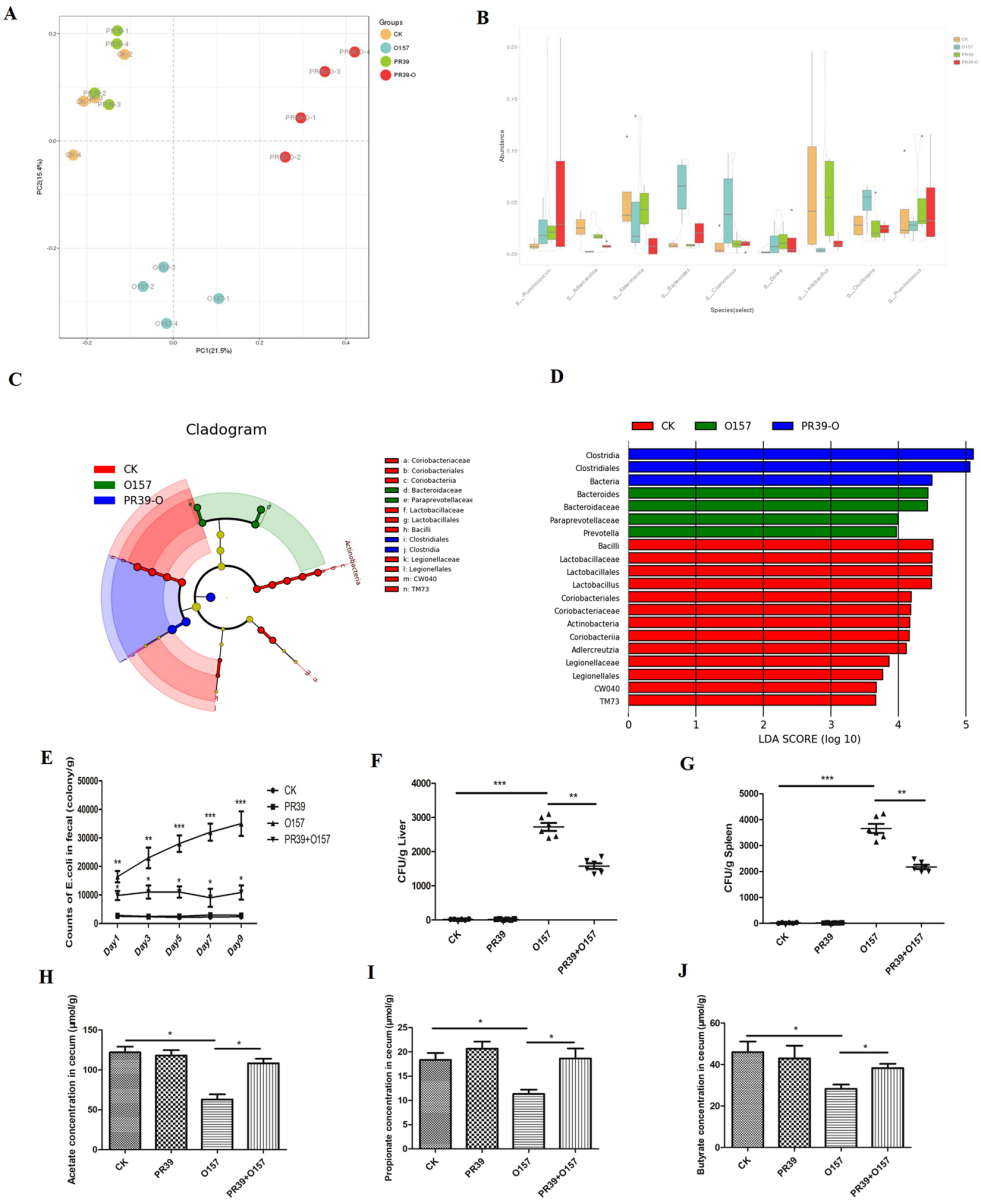
Cluster of species with similar evolutionary relationship based on Principal Co-ordinates Analysis (n = 4 per group, PCoA) (**A**). Relative abundance of the most abundant bacterial at genus level (**B**), the analysis of Ruminococcus genus based on RMG and NCBI database were displayed as g_-Ruminococcus- and g_Ruminococcus respectively. The taxonomic cladogram obtained from LEfSe analysis of 16S sequences (relative abundance ≥0.1%) (**C**). Linear Discriminant Analysis (LDA) based value compared between group CK, O157 and PR39 + O157 (**D**). The length of the histogram represented the effects size of difference bacteria and the higher score of LDA of the bacteria meaned the more dominant in the sample. Only taxa meeting an LDA scoreå 2 and P <0.05 are shown. The counts of *E*. *coli* in fecal per gram contents during each day (**E**). The colonies of *E*. *coli* in liver (**F**) and spleen (n = 6 per group) (**G**). The concentrations of acetate (**H**), propionate (**I**) and butyrate (**J**) in the cecal contents per gram determined by gas chromatography (n = 6 per group). *Means p < 0.05, **means p < 0.01, ***means p < 0.001.

Through 16 s rRNA sequencing, the difference of microbiota in cecum of each groups were analyzed. As shown in Fig. 7A, group PR39 + O157 had more similar evolutionary clusters with group CK, than group O157. The relative abundance of *Bacteroides* in other three groups were significantly lower than group O157, and the relative abundance of *Lactobacillus* in other three groups were significantly higher than group O157 (Fig. 7B). Based on the LEfSe analysis (Fig. 7C) and LDA value (Fig. 7D) confirming that *Bacteroides* were the advantage genera in group O157 among four groups. As for the counts of *E*. *coli* in feces, group O157 had highly significant colonies than group CK (p < 0.01) since day 1 and day 3, then the difference become more significant (p < 0.001), while the group PR39 + O157 had colonies only significantly higher than group CK since day 1 (p < 0.05) (Fig. 7E). As to liver and spleen tissues, the colonies of *E*.*coli* in group O157 showed significantly higher than group CK (p < 0.001), while there still existed a highly significance between group O157 and PR39 + O157 (p < 0.01) (Fig. 7F–G). The acetate, propionate and butyrate productions in cecum of group CK and PR39 + O157 were significantly higher than group O157 consistently (p < 0.05) (Fig. 7H–J).

## Discussion

*E*. *coli* O157:H7 is a notorious Shiga-toxin producing enteric pathogen. It is associated with haemorrhagic colitis outbreaks^2^, this serotype could also cause haemolytic uremic syndrome (HUS) in humans^17^. In the study, we found that the inoculation of *E*. *coli* O157:H7 significantly lowered the weight of mice, as well as survival rate, and the administration of PR39 significantly improved these trends ((Supplemental Fig. S1). Thymus and spleen indices are important factors reflecting the functional status of adaptive immunity^18^, and challenge with *E*. *coli* O157:H7 significantly lowered both, demonstrating the adaptive immunity function was partly impaired. Meanwhile, PR39 treatment recovered the levels of immunity closer to the normal state.

The intimate attachment of *E*. *coli* O157:H7 to the intestinal mucosa will cause attaching and effacing lesions^19^, which result in severe destruction of intestinal epithelium and increased intestinal permeability^6^. The result of Supplemental Fig. S2A indicated that PR39 treatment significantly attenuated the decreasing trend of TEER value in jejunum induced by *E*. *coli* O157:H7 challenge, which proving that PR39 can improve the barrier function of intestinal epithelium. Experiments investigating the serum FITC-dextran concentration also demonstrated the protective effect of PR39 on intestinal barrier function. D-lactic acid and diamine oxidase in serum were highly positively correlated with the degree intestinal damage and permeability^20^, the serum concentration of this two indexes also consistently supported that treatment with PR39 improved the abnormal increase in intestinal epithelium permeability caused by *E*. *coli* O157:H7. Because the attached bacteria in infection secrete Shiga toxin into the circulation, causing an inflammatory response in the affected cells^21^, we also found the levels of IL-1β, IL-6, TNF-α were significantly increased in infected group alone (Supplemental Fig. S3A–C), which indicated systematic inflammation in the mouse model. There was also lower levels of IL-10 in serum (Supplemental Fig. S3D), which could aggravate the inflammation. As expected, PR39 treatment significantly attenuated the level of inflammation, which was consistent with previous studies where PR39 had an anti-inflammatory immunoregulatory effect in infection^13,16^.

To evaluate the effects of PR39 on the structural and barrier function of intestinal epithelium more precisely, we further observed the morphology and villus height: crypt depth (V:C) ratio in each small intestine and colon segment (Figs 1 and 2). The small intestine, especially jejunum, is essential for the digestion and absorption of nutrients^22^, while the colon plays an important role in the reuse of small intestine surplus nutrients and cecal fermentation products^23^. In this study, the morphology of the small intestine and colon had obvious pathological characteristics including epithelium shedding in the small intestine, oedema in ileal submucosa, and structural disorder in colonic epithelium. The microscopic and submicroscopic structural observations of jejunal epithelium infected with *E*. *coli* O157:H7 showed severe damage of microvilli, which may predominantly be due to the acceleration of apoptosis *in situ* (Fig. 2C). This in turn will cause the increasing intestinal permeability observed. It has been reported that *E*.*coli* O157:H7 infection can damage the intestines, especially to the colon^24^, which were consistent with our results. Importantly, the Shiga toxin secreted by *E*. *coli* O157:H7 can pass through the impaired intestinal epithelium more easily, which will finally lead to systemic inflammation^25^, as described in Supplemental Fig. S3. There are several studies reporting that PR39 can inhibit apoptosis of inflammatory cells, HeLa cells, and kidney cells^26–28^. Correspondingly, in our study, we demonstrated that PR39 could improve the increased apoptotic index in jejunum epithelium induced by *E*. *coli* O157:H7, which consequently ameliorated the pathomorphological changes in jejunum and microvilli structure.

The intestinal mucosa is an important functional barrier against pathogen and toxin invasion^29^. Thus, impaired intestinal barrier function could lead to exposure of host to pathogens, resulting in inflammation^30^. The function of the intestinal barrier is largely determined by TJs and mucins^31,32^. TJs are intercellular structures in the intestinal mucosa which form semipermeable barriers that selectively allow substances to cross the mucosa^33^. Typical TJs include ZO-1 and occludin. Previous studies have shown reduced TJ expression in the intestine of EHEC infected mice^34^, which was verified in our study (Fig. 3). What especially interested us is that PR39 treatment increased the expression of ZO-1 and occludin, and also other TJ-related proteins. The layers of mucus mainly consist of mucins that expressed in the epithelial surface of the intestine^35^. The mucus of the major intestine segments primarily consist of mucin-1 and -2^36^, and a pathological decrease in the mucus layer in patients with ulcerative colitis is related to reduced expression of mucins^37^. MUC-2 production is also decreased in the colon of mice with dextran sodium sulphate (DSS) induced colitis^38^. In this study, we found that PR39 could attenuate the *E*. *coli* O157:H7-induced inhibition of MUC-1 and 2 gene expression in jejunum, similar to results presented in a previous study^39^.

Infiltration of inflammatory cells into submucosa of intestinal tissue is a typical feature indicating the level of inflammation^40,41^. The higher value of inflammatory invasion index means the greater number of inflammatory cells accumulating at intestinal sites, this results in continuous secretion of proinflammatory cytokines, which in turn exacerbates the inflammatory response. In this study, the inflammatory invasion index of jejunum and colon were significantly elevated in *E*. *coli* O157:H7 group, accompanied by an increased concentration of proinflammatory cytokines at the local site, as well as MPO, whose activity is positively correlated with the invasion degree of neutrophils^42^. The elevated synthesis of iNOS will lead to the raised synthesis rate of NO, which will in turn form NO reactants and thus lead to cell damage^43^. Moreover, earlier studies have shown that lowering COX-2 concentrations could reduce the risk of intestinal ulcers^44^. Additionally, iNOS acts in synergy with COX-2 to promote the inflammatory response^45^. Notably, the concentration of both INOS and COX-2 at local sites of jejunum and colon mucosa were consistently elevated after infection. In contrast, treatment with PR39 significantly attenuated the secretion of the proinflammatory factors discussed above, which may be correlated with the immunomodulatory functions of PR39^46^. Furthermore, the pathomorphological changes in the liver and kidneys induced by EHEC O157:H7 infection were largely improved by PR39 intervention (Fig. 6), this effect can probably be attributed to the increased intestinal barrier function and attenuated inflammation level in the location site as demonstrated above.

Finally, we investigated the microbiota present in the cecum contents, as the imbalance of intestinal microbiota will cause disorders of digestive function^47^, immune dysfunction^48^, abnormal development of the intestine^49^, as well as increase host susceptibility to pathogen infection^50^. The results showed that PR39 treatment led to lower levels of *Bacteroides* and higher levels of *Lactobacillus* in the cecum compared with group O157. As a whole, the microbiota composition in the group PR39 + O157 were close to the group CK, and the recovery of volatile fatty acid (VFA) production in the group PR39 + O157 also indicated that PR39 could effectively promote the balance of the microbiota in the cecum. As far as we can determine, this is the first report on the effects of PR39 on intestinal microbiota. The persistent reduction of *E*. *coli* in faeces, in addition to the reduced transference of bacteria to the liver and spleen, after PR39 treatment demonstrated that this proline-rich antimicrobial peptide had multiple functions in the intestine, mainly associated with maintaining intestinal barrier function, immunoregulation, and balancing the intestinal microbiota, not just the antibacterial effects researchers have focused on previously.

In conclusion, PR39 could treat the infection caused by EHEC O157:H7. Therefore, the porcine-derived antimicrobial peptide PR39 could be a potential drug in the treatment of EHEC O157:H7-caused intestinal disease.

## Materials and Methods

### Reagents

Antimicrobial peptide PR39 was synthesized by GL Biochem (Shanghai China), and the purity was more than 95% by reversed-phase high performance liquid chromatography analysis. the peptide was prepared in sterile saline and stored under −80 °C until used. Other normal reagents were listed on the paper we have previously published^51^, to introduce briefly: Rabbit polyclonal Abs for zonula occludens (ZO-1), Occludin, CD177 and F4/80 were purchased from Abcam (Cambridge, MA). The secondary Abs used for the immunohistochemistry was goat anti-rabbit IgG conjugated with HRP (Abcam) and for the immunofluorescence were goat anti-rabbit IgG labeling with FITC and Cy3 (Abcam), the TUNEL kit was purchased from Roche (USA). The EHEC O157:H7 ATCC43889 was purchased from China General Microbiological Culture Collection Center (Beijing, China).

### Establishment of EHEC O157:H7 induced disease model

Mice (40 in total) with average weight of 20 g were obtained from the Laboratory Animal Center of the Chinses Academy of Sciences (Shanghai, China). Just as the same feeding model we have previously carried out^51^, the mice were individually housed and maintained on a 12:12 h light-dark cycle under specific pathogen free conditions, with free access to feed and water throughout the experimental period. After five days adaptive period, the mice were randomly divided into four groups, each group had ten mice. The experiment lasted 10 days. At day 1, 5, 9, the group O157 and PR39 + O157 were orally administered 0.1 ml PBS containing 1 × 10^6^ CFU of EHEC O157:H7, while the group CK and PR39 were administered equal volume of PBS. After administration of *E*. *coli* O157 for 1 hour, the group PR39 and PR39 + O157 were intraperitoneally injected with 5.0 mg/kg PR39 dissolved in 0.25 ml sterile saline, while the group CK and O157 were administered equal volume of sterile saline. At day 10, the survival rate of each group was calculated using survival amount divided by original amount of each group. All the survived mice were euthanized at day 10. To ensure the consistency of experiment time and tested samples between each group, we finally collected six samples of each group for further analysis, for group CK, PR39 and PR39 + O157, six samples were selected randomly. The Animal Care and Use Committee of Hainan University approved all experiments, and the experimental process was conducted strictly in accordance with the Guidelines for the Care and Use of Animals for Research and Teaching at Hainan University.

### Index of immune organs

The method was according to our published work^51^, to introduce briefly, mice were sacrificed and the spleen and thymus were isolated (n = 6 per group). The blood on organ surfaces was drained with filter paper before weighing, and the immune organs index (IOX) was calculated using the following formula:

$$\mathrm{IOX}=\mathrm{weight}\,\mathrm{of}\,\mathrm{immune}\,\mathrm{organs}\,\mathrm{(mg)/body}\,\mathrm{weight}\,\mathrm{(g)}$$

### Trans epithelial electric resistance measurement in jejunum

The process to determine the value of TEER in jejunum was according to our modified method^51^, to introduce briefly, the multichannel voltage-current clamp (model VCC MC6, Physiologic Instruments) was used to determine the trans epithelial electrical potential of fresh jejunum tissues, following the protocols described previously^52,53^ and our modified method^51^. Fresh jejunum tissues were excised from mice and immediately immersed in oxygenated Krebs’s buffer, then mounted onto Ussing chambers (World Precision Instruments, Narco Scientific, Mississauga, Ontario, Canada). The Ussing chambers were equipped with two pairs of Ag/AgCl electrodes connected to the chambers via 3 M KCl/3.5% agar bridges, to measure the potential difference (PD) and passing current (I). For each test, the PD value was clamped to 20 mV, and the necessary current was recorded, the electrical resistance was determined according to Ohm’s law: [R = (20 mV − PD)/I]. To ensure comparability of TEER measurements of each group, the differences between the effective exposed area of the epithelium and the apparent exposed tissue area were normalized by presenting all measurements as a percentage of the TEER value at the end of the equilibration period for each tissue insert.

### ELISA determination

The concentrations of D-lactic acid (D-LA), diamine oxidase (DAO) and IL-10 in serum, IL-1β, IL-6, TNF-α in serum, jejunum and colon tissues were determined using ELISA kit (Boster China Wuhan), the concentrations of enzyme MPO, INOS and COX-2 in jejunum and colon were tested by ELISA kits (Keygen China Nanjing). The ELISA analysis was carried out according to the manufacturer’s instructions.

### The serum concentration of FITC-dextran determination

4 h before sampling, 250 μL of FITC-dextran was intragastrically administrated, the serum was collected and fluorescence intensity was detected with a microplate reader (Spectrumax M5 molecular devices USA).

### Scanning Electron Microscopy

Preparation of SEM samples were according to our previous published process^51^, to introduce briefly, the tissue of jejunum were fixed with 2.5% glutaradehyde for 24 h and then with 1% O_S_O_4_ for 1 h. The specimens were then dehydrated in a graded series of ethanol for 20 min at each step, then transferred into a mixture of alcohol and isoamyl acetate (V:V = 1:1) for 30 min and isoamyl acetate alone for 1 h. Finally, the specimens were then dehydrated in a Hitachi Model HCP-2 critical point dryer with liquid CO_2_, the dehydrated specimens were coated with gold-palladium and visualized using a Philips Model SU8010 FASEM (HITACHI, Japan).

### Intestinal and parenchymal organs morphology

Preparation of H&E staining samples were according to our previous published process^51,54^, to introduce briefly, intestinal tissues of the middle part of duodenum, jejunum, ileum and colon, and middle site of organs of liver and kidney were cut out to carry out hematoxylin-eosin (H&E) staining. Briefly, the above tissues from six individuals in each group were isolated, and immediately fixed in 4% paraformaldehyde solution, then embedded in paraffin, samples were sliced and stained with hematoxylin and eosin in turn. The morphological characteristics were observed with Leica NEWDM 4500BR microscope (Leica Wetzlar Germany) under certain magnifications, and the villous height and crypt depth were measured using image pro software (MediaCybernetics MD USA).

### Immunofluorescence analysis

Preparation of immunofluorescence samples were according to our previous published process^51^, to introduce briefly, the tissues of jejunum of each groups were isolated and fixed in 4% paraformaldehyde, then embedded with paraffin and sliced for immunofluorescence analysis. Briefly, sections of 5 mm thickness were deparaffinized and rehydrated and proceed antigen retrieval. The sections were then incubated in 3% hydrogen dioxide for 20 min without light. Then the sections were incubated with primary antibodies (1:200 dilution) specific for ZO-1 and Occludin (Abcam USA). TRITC-conjugated goat anti rabbit IgG for ZO-1 and FITC- conjugated goat anti rabbit IgG for Occludin (JIR USA) were added at a ratio of 1:100 and left to be incubated at room temperature for 1 h in darkness. DAPI was then used to stain the nucleus. Glycerol was used to mount the samples onto slides. Images were taken under Leica fluorescence microscope (Keyence, Osaka, Japan).

### Immunohistochemistry analysis

Preparation of immunohistochemistry samples were according to our previous published process^51^, to introduce briefly, for immunohistochemical analysis of CD177 and F4/80 of jejunum and colon, nonspecific binding sites were blocked with PBS containing 1% w/v BSA for 30 min, anti-CD177 and F4/80 antibodies (Santa USA) were added at a dilution of 1:100 and incubated overnight at 4 °C. Samples were washed four times in PBS and treated with HRP-conjugated rabbit anti goat IgG (JIR USA) at a ratio of 1:100, then samples were incubated at 4 °C for 1 h and washed with PBS three times. DAB (DAKO USA) was added then hematoxylin was used to counterstain the slices. The samples were dewarerd with gradient alcohol, and xylene was used to increase the transparency of slides, a neutral balsam was applied for mounting. For evaluating the apoptosis level of jejunum tissues, paraffin sections were dewaxed with water and antigen retrieval was executed. TdT and dUTP (Roche USA) were mixed at a ratio of 1:9 and incubated at 37 °C for 60 min, the endogenous peroxidase was blocked, and the slides were allowed to dry. The tissue was then covered with converter-POD (Roche USA) and incubated at 37 °C for 30 min, and washed with PBS three times. DAB was added to the slices, and distilled water was used to stop color development. Finally, the cell nucleus was counterstained and the slices were dehydrated and mounted.

### Intestinal microflora analysis based on 16 s rRNA sequencing

The cecum microbiota sequencing based on 16 s rRNA were carried out according to our previous published work^51^, to introduce briefly, Total Genomic DNA from cecal contents of four mice in each group were extracted and V4–V5 regions of bacterial 16S rRNA gene were amplified, and further performed by Illumina Hiseq PE250 (SAGENE, Guangzhou China). Genomic DNA was extracted from the cecal contents of mice using fecal DNA kit (Sigma-Aldrich, Germany) according to the manufacturer’s instructions. The purity and quality of DNA were quantified using a Nanodrop 2000 spectrophotometer. V4-V5 regions of bacterial 16S rRNA gene (from 507 to 907) were amplified from extracted DNA using bar-coded primers 515 F (5′-GTGCCAGCMGCCGCGG-3′) and 907 R (5′- CCGTCAATTCMTTTRAGTTT-3′). The original data were filtered through removing reads containing more than 10% of unknown nucleotides or containing less than 80% of bases with quality (Q-value)å 20, the filtered reads were then assembled into tags according to overlap between paired-end with more than 10-bp overlap and less than 2% mismatch. The high-quality sequences were clustered into operational taxonomic units (OTUs) defined at 97% similarity. Taxonomic assignments of OTUs were made using quantitative insights into microbial ecology (QIIME) software^55^ through comparison with the databases of SILVA^56^, Greengene^57^ and RDP^58^. The distribution of OTUs at different taxonomic levels were shown as box plot, and the principal component analysis (PCA) were plotted based on the relative abundance of OTUs in each group. Linear discriminant analysis (LDA) effect size (LEfSe) method was used to identify the most differentially abundant taxons between groups^59^. The cladogram analysis was conducted based on LEfSe analysis of 16S sequences (relative abundance ≥0.1%), and the cladogram plot was drawn through the software of Fig Tree (v1.4.4, http://tree.bio.ed.ac.uk/software/figtree/). Statistical Analysis of Metagenomic Profile package (STAMP, http://kiwi.cs.dal.ca/Software/STAMP) was used to compare the difference between Groups.

### Gas chromatographic analysis

Concentration of short-chain fatty acids (SCFAs) in the cecal contents of mice were determined using GC-8A gas chromatography (Shimadzu, Kyoto, Japan). Briefly, the cecal content (n = 6 per group) were weighed and mixed with 1 ml distilled H_2_O, centrifuging at 12000 g at 4 °C for 15 min. The supernatant was collected and mixed with 85% orthophosphoric acid for 1 h at 4 °C, after centrifuging at 12000 g at 4 °C for 15 min, the supernatant was collected and transferred into the gas chromatography vial to detect SCFA concentrations by GC-8A, finally, the SCFA concentrations were normalized to the weight of the cecal contents.

### Bacterial shedding and translocation analysis

The bacterial shedding and translocation analysis was conducted according to our previous procedure^51^, to introduce briefly, the feces were collected at day 1, 3, 5, 7 and 9, (n = 6 per group). The bacterial shedding status were determined by plating series dilutions on Eosin Methylene Blue (EMB) agar plates (Hopebio China). At day 10, mice were sacrificed, and the spleens and livers were removed aseptically from six mice of each groups, homogenized in 4 °C cold PBS, the numbers of CFU were determined by plating series dilutions on EMB agar plates.

### Real-time PCR for gene expression analysis

The qRT-PCR analysis procedure was carried out referring to our published work^54^, to introduce briefly, RNA samples (n = 6 per group) were extracted from the middle jejunum tissues, total RNA isolation and cDNA synthesis by reverse transcription were conducted using Trizol reagent (Invitrogen Carlsbad CA USA) and M-MuLV reverse transcriptase kit (Fermentas EU Glen BURNIE USA) respectively. The mRNA levels of individual genes were measured by real-time PCR using the SYBR Premix Ex Taq Kit (Takara Biotechnology Shiga Japan) in the ABI Step One Plus Real-Time PCR system (Applied Biosystems CA USA), data was analyzed according to the comparative threshold cycle (Ct) method and normalized to an endogenous reference GAPDH. The related primers used in the experiment were listed in Table 1, the relative expression levels of barrier function related genes (ZO-1, ZO-2, claudin-1, occluding, mucin-1, mucin-2) were analyzed on jejunum tissues.

**Table 1:** Sequences of primer for qRT-PCR analysis.

Gene	Product size (bp)	Sequence (5′ → 3′)	Annealing temperature(°C)	accession number
GAPDH	212	F: GAGAAACCTGCCAAGTATGATGAC R: TAGCCGTATTCATTGTCATACCAG	57	NM_008084.3
ZO-1	198	F: TCATCCCAAATAAGAACAGAGC R: GAAGAACAACCCTTTCATAAGC	**57**	XM_006540786.1
ZO-2	269	F: GCTTTGGTGTGGACCAAGAT	60	XM_006526909.1
Claudin-1	110	R: TCCATTATGGGTTTGCATGA F: GCTGGGTTTCATCCTGGCTTCT R: CCTGAGCGGTCACGATGTTGTC	**58**	NM_016674.4
Occludin	86	F: CTTTGGCTACGGAGGTGGCTAT R: CTTTGGCTGCTCTTGGGTCTG	58	XM_006517566.1
Mucin1	147	F: TGGATTGTTTCTGCAGATTTT R: CCTGACCTGAACTTGATGCT	**60**	NM_013605.2
Mucin2	134	F: CCCAGAAGGGACTGTGTATG R: TGCAGACACACTGCTCACA	60	NM_023566.3

### Statistical analyses

Multiple comparison test were carried out by Tukey’s HSD and one way analysis of variance (ANOVA) with SPSS 18.0 (SPSS Chicago IL), a value of p < 0.05 was considered as significant, a value of p < 0.01 was considered as highly significant, results are expressed as mean ± SD.

### Accession numbers of nucleotide sequence

Original data of this project have been deposited into the NCBI nucleotide database under accession number PRJNA522846, the exact information about the relative percentage at different taxonomic levels was provided as Supplemental Table S1.

## Supplementary information

Supplementary information

Supplemental Table S1
