# Supplementary material for: Cathelicidin- derived PR39 protects enterohemorrhagic Escherichia coli O157:H7 challenged mice by improving epithelial function and balancing the microbiota in the intestine
Source: Sci Rep. 2019 Jul 1;9:9456. doi: 10.1038/s41598-019-45913-6 (PMC6603261; doi:10.1038/s41598-019-45913-6)
Supplement: Supplementary file 1 — Supplementary information [file 41598_2019_45913_MOESM1_ESM.pdf]

Cathelicidin- derived PR39 protects enterohemorrhagic *Escherichia coli* O157:H7 challenged mice by improving epithelial function and balancing the microbiome in the intestine

Zhang Haiwen[12], Hua Rui[1], Zhang Bingxi[1], Guan Qingfeng[12], Wang Beibei[*2], Zeng Jifeng[12], Wang Xuemei[12], Wu Kebang[*12]

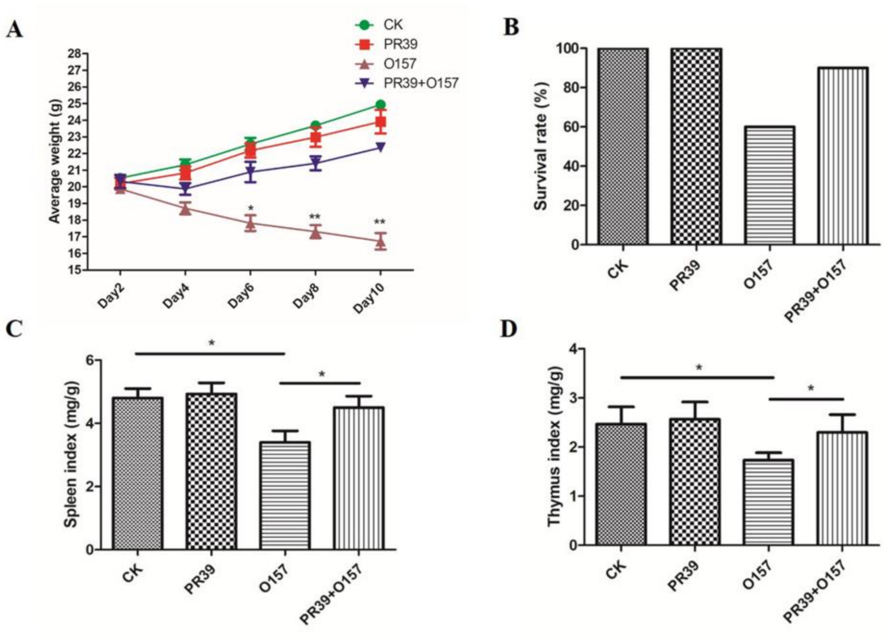

Supplemental Figure S1**.** Body weight of mice in each group (A) and finally survival rate at day 10 (B). Spleen index (C) and thymus index (D) of mice (n=6) in each group. All of the data are expressed as the mean ± SD. * means $p < 0.05$ compared with CK group, ** means $p < 0.01$ compared with CK group.

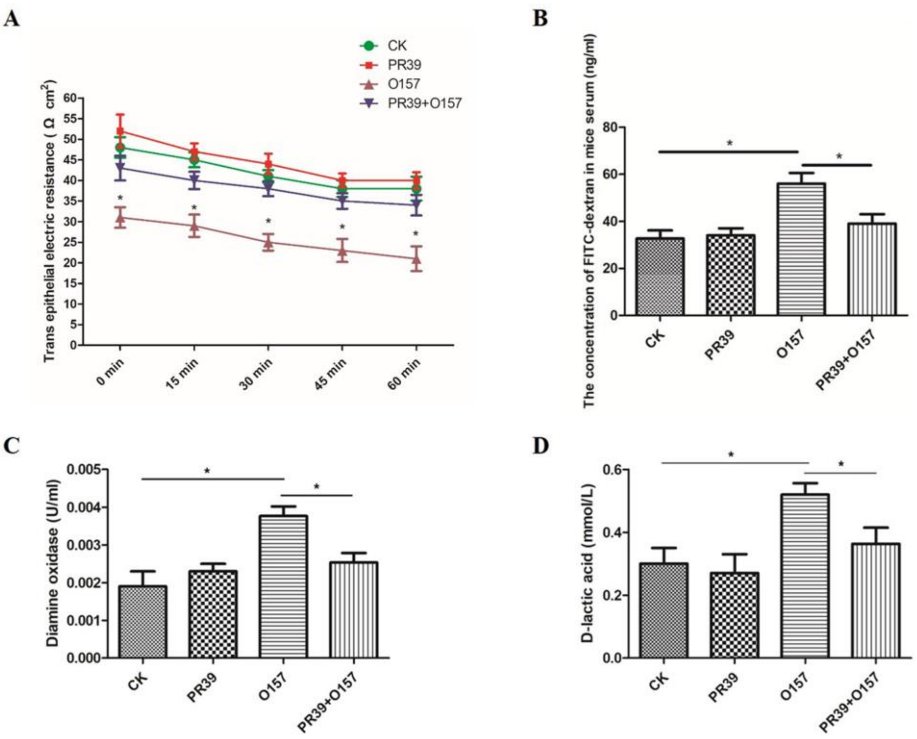

Supplemental Figure S2**.** Dynamic change (0-60 min) of TEER value in jejunum epithelium (n=5) (A). Concentration of FITC-dextran in serum of mice (n=6) (B). Diamine oxidase (C) and D-lactic acid (D) in serum of mice (n=6). All of the data are expressed as the mean ± SD. * means $p < 0.05$, # means $p < 0.01$ compared with CK group.

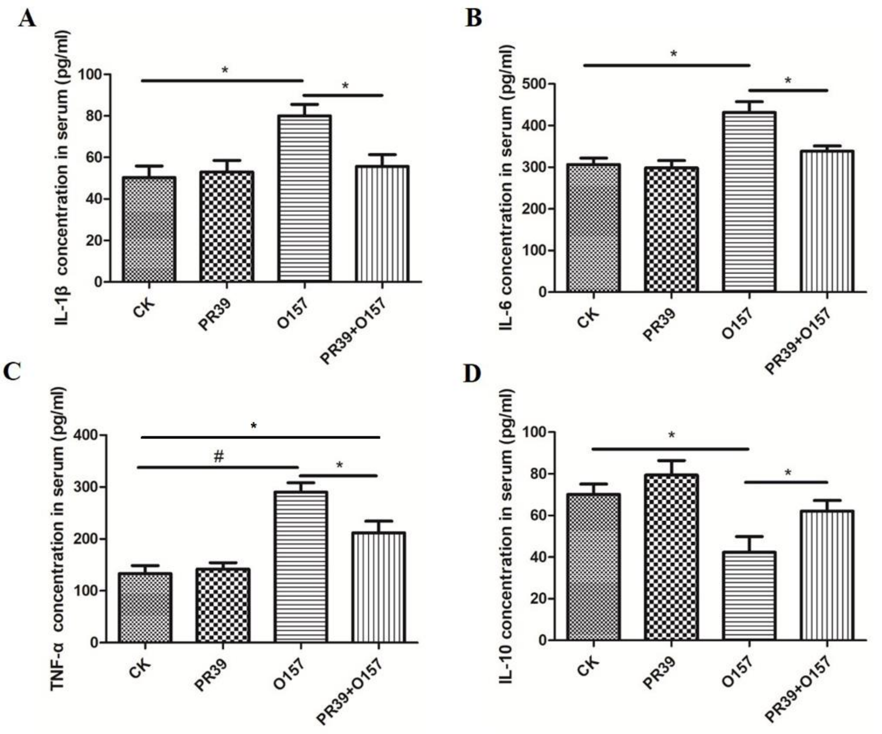

Supplemental Figure S3. Concentration of IL-1β (A), IL-6 (B), TNF-α (C), IL-10 (D) in serum, there were six repetitions (n=6) in each group and the average values were calculated. All of the data are expressed as the mean ± SD. * means $p < 0.05$, # means $p < 0.01$ compared with CK group.
